# DNA Nanostructures and DNA‐Functionalized Nanoparticles for Cancer Theranostics

**DOI:** 10.1002/advs.202001669

**Published:** 2020-10-15

**Authors:** Fay Nicolson, Akbar Ali, Moritz F. Kircher, Suchetan Pal

**Affiliations:** ^1^ Department of Imaging Dana‐Farber Cancer Institute & Harvard Medical School Boston MA 02215 USA; ^2^ Center for Molecular Imaging and Nanotechnology Memorial Sloan Kettering Cancer Center New York NY 10065 USA; ^3^ Department of Chemistry Indian Institute of Technology‐ Bhilai Raipur Chhattisgarh 492015 India; ^4^ Department of Radiology Brigham and Women's Hospital & Harvard Medical School Boston MA 02215 USA

**Keywords:** cancer, DNA‐nanostructures, nanoparticles, theranostics

## Abstract

In the last two decades, DNA has attracted significant attention toward the development of materials at the nanoscale for emerging applications due to the unparalleled versatility and programmability of DNA building blocks. DNA‐based artificial nanomaterials can be broadly classified into two categories: DNA nanostructures (DNA‐NSs) and DNA‐functionalized nanoparticles (DNA‐NPs). More importantly, their use in nanotheranostics, a field that combines diagnostics with therapy via drug or gene delivery in an all‐in‐one platform, has been applied extensively in recent years to provide personalized cancer treatments. Conveniently, the ease of attachment of both imaging and therapeutic moieties to DNA‐NSs or DNA‐NPs enables high biostability, biocompatibility, and drug loading capabilities, and as a consequence, has markedly catalyzed the rapid growth of this field. This review aims to provide an overview of the recent progress of DNA‐NSs and DNA‐NPs as theranostic agents, the use of DNA‐NSs and DNA‐NPs as gene and drug delivery platforms, and a perspective on their clinical translation in the realm of oncology.

## Graphical abstract

DNA‐based artificial nanomaterials, namely, DNA nanostructures (DNA‐NSs) and DNA‐functionalized nanoparticles (DNA‐NPs), are emerging as an all‐in‐one platform for imaging and therapy of different cancers. The ease of attachment of both imaging and therapeutic molecules, biostability, biocompatibility has augmented the growth of the field. This review aims at providing an overview of the recent progress in this rapidly expanding field.

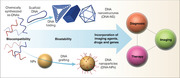

## 1 Introduction

Cancer is the first or second leading cause of death in humans below the age of 70 in over 50% of countries.^[^ ^1^ ^]^ More alarmingly, the annual number of new cancer cases is projected to grow from 18.1 million in 2018 to 29.4 million in 2040 due to population growth, aging, and increasing pollution.^[^ ^1a^ ^]^ Currently, cancer treatment options for solid tumors include removal of cancerous tissue via surgery, chemotherapy, radiation therapy, immunotherapy, or a combination of some or all of these approaches. Once a solid tumor without the presence of metastases is diagnosed, the primary line of treatment in most cases is surgery,^[^ ^2^ ^]^ which improves the clinical outcome in the majority of cases.^[^ ^3^ ^]^ However, complete surgical removal of cancer tissue is often not feasible due to i) poor visual contrast between cancerous and healthy tissue and ii) the fact that surgeons are often hindered in resecting the full tumor extent because critical structures such as nerve and blood vessels run near or through the diseased tissue. To alleviate this issue, molecular imaging agents are used to augment the contrast between healthy and diseased tissues,^[^ ^4^ ^]^ However, current molecular imaging techniques have significant drawbacks in detecting the true microscopic extent of cancer.^[^ ^4^, ^5^ ^]^ In addition, conventional chemotherapeutic agents are relatively nonselective, causing frequent severe side effects which can range from acute manifestations to chronic problems that deteriorate the quality of life of cancer patients. Other limitations of chemotherapy include the development of drug resistance.^[^ ^6^ ^]^ It is therefore desirable to develop anticancer drugs that can target cancer cells in a more selective manner, thus reducing side effects while improving therapeutic efficacy. Taken together, the main goals for better cancer treatment and mitigation are: i) early diagnosis, ii) complete surgical removal of the macroscopic tumor burden, and iii) selective delivery of anticancer drugs to destroy residual cancerous tissues.

With regards to cancer, the ideal therapy would deliver the correct treatment to the specific target in a localized and controlled manner, and in turn, minimize systemic side effects.^[^ ^7^ ^]^ Of course, this is an extremely challenging task, namely, due to tumor heterogeneity between patients. However, in recent years, the use of theranostics has emerged as a promising means to overcome such challenges.^[^ ^8^ ^]^ Theranostics combines diagnostics with therapeutics, enabling cancer to be diagnosed and treated on an individual, personalized level, and allowing the therapeutic efficacy to be monitored noninvasively and in real time.^[^ ^9^ ^]^ Such platforms utilize contrast agents which can be tracked in vivo using at least one imaging modality, e.g., magnetic resonance imaging (MRI),^[^ ^10^ ^]^ positron emission tomography (PET),^[^ ^11^ ^]^ computed tomography (CT),^[^ ^12^ ^]^ surface‐enhanced Raman scattering (SERS),^[^ ^13^ ^]^ or fluorescence.^[^ ^14^ ^]^ A therapeutic module such as chemotherapeutics,^[^ ^15^ ^]^ immunotherapies,^[^ ^16^ ^]^ or photosensitive molecules^[^ ^17^ ^]^ is also incorporated. Nanotheranostics makes use of nanomaterials, e.g., proteins, polymers, or DNA as a host material for imaging and therapeutic agents. In recent years, substantial efforts have resulted in the production of a range of nanoplatforms with theranostic capabilities.^[^ ^7^, ^18^, ^19^ ^]^ Nanotheranostics have been explored extensively in oncology for applications, including the monitoring of intratumoral drug delivery,^[^ ^20^ ^]^ image‐guided focal therapy,^[^ ^21^ ^]^ and monitoring of changes in the tumor microenvironment (TME) as a response to therapy.^[^ ^22^ ^]^

Following treatment, the optimum nanotheranostic should, therefore, be thought of as a platform capable of providing long‐term, patient‐specific information on disease status. As such, when designing nanotheranostics, researchers must go above and beyond to create a platform that offers several advantages over traditional approaches, a key requirement that will further support the translation of nanotheranostics into the clinic. This can be achieved by taking advantage of and incorporating several unique functionalities inherent to nanomaterials in order to deliver personalized medicine. Such features include: i) rapid and highly selective accumulation within the tumor; ii) the reporting of biochemical and morphological changes from the region of interest; iii) delivery of an effective therapeutic response; (iv) safe and biodegradable properties; and (v) offering a benefit over traditional treatments by improving drug efficacy and consequently patient tolerance.^[^ ^23^ ^]^ Therefore, owing to the desire to incorporate all of these necessities, the design of nanotheranostic agents is far from simple.

It is well established that blood vessels in malignant lesions are more permeable to macromolecules (size range of >10 nm) than those in healthy tissues, and these molecules are subsequently retained within the tumor tissue due to impaired lymphatic clearance. This phenomenon is referred to as “enhanced permeability and retention” (EPR) effect or “passive targeting.”^[^ ^24^ ^]^ Macromolecules and NPs that are surface‐functionalized with moieties targeting the cancer microenvironment or cancer cells can further enhance the intratumoral homing, which is termed as “active targeting.”^[^ ^25^ ^]^ Further, innovative strategies have also been developed to integrate anticancer drug or drug combinations in the nanostructures via covalent and noncovalent chemistry.^[^ ^26^ ^]^ In the last decade, a better understanding of tumor biology and synthesis of versatile nanomaterials such as polymers and polymer gels, lipids, inorganic NPs, and biomacromolecular scaffolds has aided the development of novel theranostic platforms for cancer.^[^ ^11^, ^14^, ^21^, ^27^ ^]^ The development of such materials is very diverse and extensively reviewed elsewhere.^[^ ^28^ ^]^

One of the significant drawbacks of using engineered nanomaterials is the systemic and immune toxicity, which raises significant concerns for real‐life clinical applications.^[^ ^29^ ^]^ As an alternative strategy, the last 20 years have seen enormous interest in the use artificial (synthetic) DNA‐based nanomaterials.^[^ ^30^ ^]^ DNA, a genetic material, possesses high biocompatibility and low cytotoxicity, which is optimal for biomedical applications.^[^ ^31^ ^]^ Other advantages of DNA‐based nanostructures are predictable intermolecular interaction and recognition properties; intrinsic nanoscale size (2 nm in diameter and ≈0.3 nm/per base pair); ease of chemical/enzymatic synthesis of specific sequences; and optimum half‐life in biological fluids. Further, selective chemical modifications of DNA structures with anticancer drugs and imaging modalities provide flexibility in the design of theranostic nanoprobes.^[^ ^32^, ^33^ ^]^ In this review, we present recent applications of two different artificial DNA nanostructures, namely, DNA nanostructures (DNA‐NSs) and DNA‐functionalized nanoparticles (DNA‐NPs), for the purpose of cancer theranostics. In Section 2.1, we provide a brief introduction to DNA‐NSs and DNA‐NPs. In Section 2.2, we cover the biological stability of DNA‐NSs and DNA‐NPs. We summarize recent developments in the use of DNA‐NPs and DNA‐NSs in theranostic applications in Sections 2.3 and 2.4, respectively, and specifically review the current status of DNA‐NSs and DNA‐NPs as drug and gene delivery platforms in Sections 2.5 and 2.6, respectively. In Section 3, we discuss the clinical translation of DNA‐NS and DNA‐NPs and conclude in Section 4 with our final thoughts on the future of the field.

## 2 Introduction and Applications of DNA Nanostructures and DNA‐Functionalized Nanoparticles for Cancer Theranostics

### 2.1 Brief Introduction to DNA Nanostructures (DNA‐NSs) and DNA‐Functionalized Nanoparticles (DNA‐NPs)

We thematically classify artificial DNA‐NSs into DNA tile‐based and DNA origami‐based nanostructures. Tile‐based structures are self‐assembled by hybridizing multiple unique DNA single strands. Further, hierarchical assembly via “sticky end” cohesion emanates large DNA structures. In 1983, Kallenbach et al. proposed and reported the first artificial, immobile four‐way junction, also known as Holliday junction, which is transiently formed during genetic recombination.^[^ ^34^ ^]^ Using a similar strategy, the same group constructed three‐, five‐, six‐, eight‐, and 12‐way junctions.^[^ ^35^ ^]^ However, due to substantial structural flexibility, the multi‐arm junctions were not amenable to produce higher‐order nanoscale structures. To alleviate this issue, more robust DNA double‐crossover (DX) DNA‐NSs were developed.^[^ ^36^ ^]^ In 1998, the first example of a large‐scale, 2D DNA structure was fabricated using sticky‐end cohesion two‐arm DX tiles and visualized by an atomic force microscope (AFM). This work opened up many more design opportunities and several tile‐based DNA‐NSs with diverse and intricate patterns, with or without defined boundaries, have been assembled.^[^ ^36^, ^37^ ^]^ Concurrently, several cage‐like polyhedral objects have been constructed from fixed numbers of DNA tiles and using other design principles.^[^ ^38^ ^]^ The DNA tile‐based designs lead to highly periodic and symmetric structures and therefore are not amenable to constructing nanostructures of arbitrary and well‐defined sizes and shapes. In a complementary approach, a single‐stranded DNA (ss‐DNA) tile (SST)‐based DNA‐NS, also referred to as a “DNA brick” was developed.^[^ ^39^ ^]^ In this design principle, each ss‐DNA is defined by unique sequences located in specific locations of a 2D or 3D nanoobject. Therefore, any particular DNA strand can be excluded or vice versa, thus enabling one to build a library of DNA objects with arbitrary sizes, shapes, and surface features in a high throughput manner.

**Figure 1:**
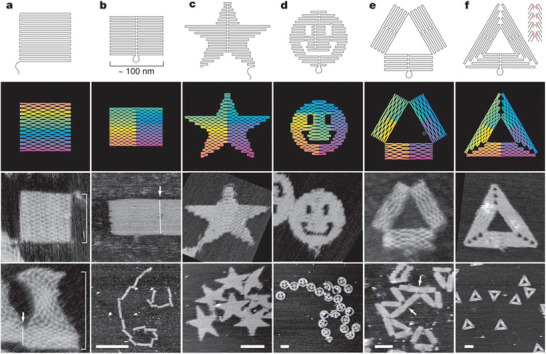
Different nanoscale shapes developed by DNA origami technology with dangling curves and loops representing unfolded sequences (top row). Diagrams demonstrate the bends at, and away, from crossovers (second row). Colors illustrate the base‐pair index along the folding path where red refers to the 1st base and purple refers to the 7000th base. AFM images of the DNA origami structures are also shown (third row). All image are the same size of 165 nm × 165 nm. Reproduced with permission.^[^ ^40^ ^]^ Copyright 2006, Springer Nature.

**Figure 2:**
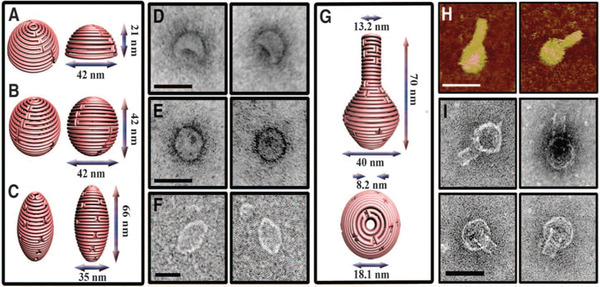
DNA origami nanostructures with complex 3D curvatures. Schematic representations of the hemisphere and corresponding transmission electron microscope (TEM) images of a,d) the hemisphere, b,e) the sphere, and c,f) the ellipsoid. Associated scale bars for the TEM images in (d)–(f) are 50 nm. g) Schematic representation of the nanoflask with expected dimensions. h) AFM images of the nanoflask in which the scale bar represents 75 nm. i) Negatively stained TEM images of the nanoflask, scale bar is 50 nm. Reproduced with permission.^[^ ^41c^ ^]^ Copyright 2011, American Association for the Advancement of Science.

**Figure 3:**
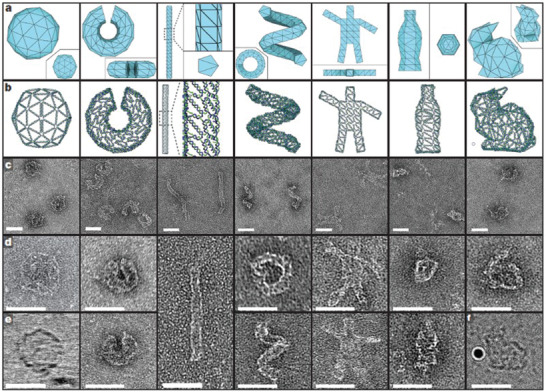
3D meshes rendered in DNA with different views of the 3D meshes serving as starting points for the initial design process. a) 3D meshes of different structures. b) The front face of each design. Single DNA strands are rendered as tubes. c–e) Negative‐stain dry‐state TEM images (except for the ball and bunny in (e) and (f), respectively) of each of the structures. Scale bars are as follows: c) 250 nm × 250 nm views; d,e) 100 nm × 100 nm close‐ups, with the exception of the pentagonal rod (200 nm × 100 nm); e,f) (ball and bunny) are imaged using cryo‐electron microscopy. The Au particle used for alignment is shown in (f). In (f), the scale bar represents 50 nm. Reproduced with permission.^[^ ^43c^ ^]^ Copyright 2015, Springer Nature.

In 2006, Rothemund developed the “DNA origami” technology, which transformed the landscape of DNA‐NSs (**Figure** **1**).^[^ ^40^ ^]^ The DNA origami uses hundreds of short ss‐DNA (staple strands) to fold a several thousand bases long ss‐DNA (scaffold strand). Rothemund used the genomic ss‐DNA from the M13 bacteriophage (7249 nucleotides long) as a scaffold strand and designed a set of ss‐DNA or “staple strands” which selectively hybridized to unique locations of the scaffold, folding it into a 2D nanoscale shape with a nearly quantitative yield of formation (Figure 1). Consequently, numerous research groups successfully extrapolated DNA origami technology to fabricate 3D objects with shapes, sizes, designer twists, and curvatures (**Figure** **2**).^[^ ^41^ ^]^ Higher‐order or larger DNA origami structures were also developed by several groups by using methods such as edge‐to‐edge base‐stacking interactions, sequence‐specific sticky end cohesion, DNA tiles, and utilization of longer scaffolds.^[^ ^42^ ^]^ In recent developments, various research groups have devised both simple and complex wireframe architectures to create DNA origami structures of complex 3D shapes (**Figure** **3**).^[^ ^43^ ^]^

**Figure 4:**
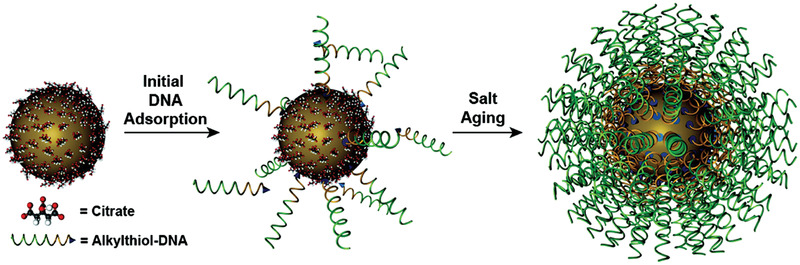
Synthesis of a DNA‐NP. A high‐density DNA‐NP is formed by using citrate‐stabilized particles incubated in alkylthiol‐functionalized DNA in water which consequently forms a low‐density monolayer. When the NPs are incubated in aqueous solutions with successively higher concentrations of salt (typically 0.15−1.0 m) and surfactants over ≈12 h, successful formation of a high‐density DNA‐NP shell is achieved. Reproduced with permission.^[^^30c]^ Copyright 2012, American Chemical Society.

DNA‐functionalized NPs (DNA‐NPs) were first reported in 1996 by Mirkin et al. in which the authors described a synthetic strategy that enabled the preparation of nucleic acid−NP nanostructures consisting of densely functionalized and highly oriented DNA covalently attached to the core surface of gold NPs (AuNPs) (**Figure** **4**).^[^ ^30^, ^44^ ^]^ DNA molecules with a thiol end were grafted onto the AuNP surface, giving rise to different properties such as a cooperative binding and subsequent sharp melting transitions, and resistance to nuclease degradation in comparison to the free DNA molecules.^[^ ^45^ ^]^ Subsequently, the core was replaced by various inorganic and polymeric materials such as Ag, Pd, Fe_3_O_4_, quantum dots, nanoshells, proteins, and polymers with distinct optical, catalytic, and physicochemical properties.^[^ ^46^ ^]^ Strategies to remove the core material to form a hollow structure surrounded by an ss‐DNA shell were also developed to improve biocompatibility.^[^ ^47^ ^]^ More recently, elegant strategies to graft DNA onto metal‐organic framework nanoparticles have been devised.^[^ ^48^ ^]^ Li et al. reported an extremely simple approach for grafting DNA onto lanthanide‐doped up‐conversion nanoparticles.^[^ ^49^ ^]^ Direct coordination of DNA with metal ions such as Fe^2+^ resulted in spherical metal‐DNA nanostructures for drug and gene delivery applications.^[^ ^50^ ^]^ In parallel, DNA‐lipid/polymer amphilites have been explored for self‐assembled nanostructures of various shapes with DNA molecules protruding out.^[^ ^51^ ^]^ Further, DNA hybridization‐based strategies have been established to integrate DNA‐NSs and DNA‐NPs structures in a single nanoscale structure with superior/tuneable optical properties and novel functionalities.^[^ ^52^ ^]^

### 2.2 Biological Stability and Cellular Uptake of DNA‐NSs and DNA‐NPs

**Figure 5:**
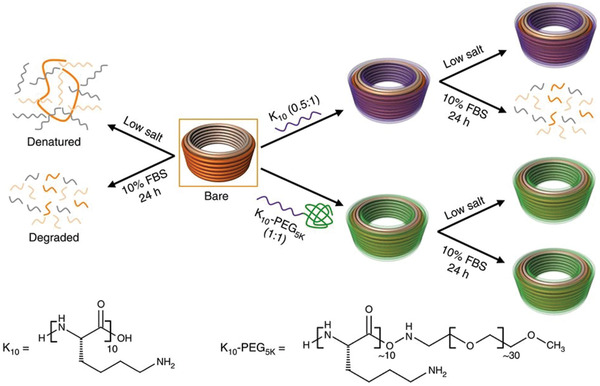
Schematic illustration of the fate of bare and coated DNA‐NS in buffers (physiological pH) at 37 °C containing either low salt or 10% fetal bovine serum (FBS). At low salt concentrations, bare DNA‐NSs rapidly become denatured and degraded in cell medium containing 10% FBS. However, it is possible to override low‐salt‐induced denaturation and nuclease degradation through coating the DNA‐NSs with various ratios of positively charged peptides (K10 or K10–PEG5K). Reproduced under the terms of the Creative Commons Attribution License.^[^ ^65^ ^]^ Copyright 2017, Springer Nature.

For a nanomaterial to be considered as a viable theranostics agent, it is essential to demonstrate biocompatibility and it should, therefore, maintain long‐term structural integrity in different physiological environments experienced on the path to cancerous sites so that no premature functional activation occurs. Thus, both DNA‐NSs and DNA‐NPs should exhibit a suitable half‐life. There has been increasing interest in understanding the structural stability of the nanostructures and fabrication of materials with enhanced stability.^[^ ^53^ ^]^ In general, artificial DNA nanostructures show enhanced stability compared to single‐stranded and double‐stranded DNA in nuclease‐containing environments.^[^ ^54^ ^]^ The last decade has seen many investigations that have aimed at assessing the biostability of DNA‐NSs. Correct folding and formation of 3D DNA nanostructures require high concentrations of divalent cations (such as ≈5 × 10^−3^–20 × 10^−3^ m of Mg^2+^), which aids in reducing the repulsion of the negatively charged phosphate backbone of DNA.^[^ ^41a,c^ ^]^ Therefore, most complex DNA‐NSs suffer from poor structural integrity in physiological conditions, which are associated with low amounts of bivalent cations. On the other hand, nuclease enzymes lead to a degradation of DNA‐NSs upon incubation under physiological conditions.^[^ ^55^ ^]^ Mei et al. reported that 2D DNA origami nanostructures were more stable in cell lysates compared to natural single‐ and double‐stranded DNA^[^ ^56^ ^]^ and all wire‐frame DNA origami‐based structures have been shown to exhibit high stability in biological milieus.^[^ ^43c,d^ ^]^ Walsh et al. demonstrated that DNA‐NSs remain stable for at least 48 h after their cellular internalization.^[^ ^57^ ^]^ Shen et al. demonstrated that the complete degradation of DNA origami nanotubes required at least 60 h using a double‐stranded DNA‐selective fluorescent probe.^[^ ^58^ ^]^ Further, simple end‐modification of DNA strands in DNA‐NSs significantly enhanced the serum stability.^[^ ^59^ ^]^ Recently, Surana et al. developed a quantitative fluorescence‐based assay to investigate structural stability and lifetime of DNA‐NS in the coelomocytes of the multicellular organism *C. elegans*.^[^ ^60^ ^]^ Using noninvasive PET, Jiang et al. evaluated the stability and biodistribution of DNA origami DNA‐NSs in vivo. Interestingly, the authors discovered that DNA origami structures accumulate in the kidney of healthy mice.^[^ ^61^ ^]^ One of the advantages of DNA‐NSs is the ease of structural modifications, which significantly enhances their structural stability. Several approaches have been investigated to alleviate stability concerns. Cassinelli et al.  developed a click chemistry‐based approach to lock DNA helices of DNA‐NSs, therefore enhancing the structural stability at low ionic strength.^[^ ^62^ ^]^ Methods to coat DNA‐NSs with cationic capsid proteins or with cationic polymers have been reported.^[^ ^63^ ^]^ Perrault et al. reported an elegant virus‐inspired encapsulation strategy of DNA‐NSs in liposomes, and therefore preventing the structure from nuclease degradation and increasing the time of circulation in vivo .^[^ ^64^ ^]^ Ponnuswami et al. demonstrated that DNA‐NSs coated with oligolysine, a positively charged polymer, exhibited enhanced stability in low salt concentrations and increased resistance to DNase I digestion compared to the uncoated counterpart (**Figure** **5**).^[^ ^65^ ^]^ More recently, glutaraldehyde‐mediated crosslinking of the oligolysines has been shown to enhance the structural stability of the DNA‐NSs up to 2 weeks in DNase I condition.^[^ ^66^ ^]^

DNA‐NPs have also been shown to possess enhanced nucleic acid stability in cellular environments by limiting the extent of degradation caused by endogenous nucleases. The dense negatively charged DNA layer and unique shape are attributed to minimal enzymatic nucleic degradation and innate immune response.^[^ ^45^, ^67^ ^]^ Patel et al. demonstrated that Dicer and serum nucleases have a lesser favorability for blunt duplexes compared to those with 3′ overhangs of DNA‐NPs. Genome‐wide expression profiling showed that DNA‐NPs induce a minimal biological response in HeLa cells. The high in vivo stability was attributed to the structural complexity of the DNA‐NPs, which diminished the accessibility and activity of nucleases. Recently, Chou et al. demonstrated an elegant strategy for the fabrication of superstructures of DNA‐NPs for reduced macrophage sequestration and to improve tumor accumulation and elimination.^[^ ^68^ ^]^ Nucleic acids are associated with a high negative charge that hinders the translocation across negatively charged lipid bilayers.^[^ ^69^ ^]^ Typically, cationic polymers such as polyethylenimine are often employed as transfection agents due to the capability of binding and condensing large nucleic acids into nanosized structures, thus aiding effective cellular uptake of nucleic acids. In contrast, DNA‐NPs are taken up very efficiently by almost all cell types without any transfection agents.^[^ ^47^, ^70^ ^]^ However, since artificial DNA‐NSs and DNA‐NPs usually carry a negative charge, passive uptake into the cells is not feasible due to the electrostatic repulsion and they are instead, internalized by an active energy‐dependent transport pathway. Liang et al. demonstrated that tetrahedral DNA‐NSs are uptaken by a caveolin‐dependent pathway, and thereafter, are transported to the lysosomes using microtubules.^[^ ^71^ ^]^ Aptamer‐modified DNA‐NSs were shown to be internalized by the surface receptors and compartmentalized in endosomes and subsequently in acidic lysosomes.^[^ ^72^ ^]^ There is a significant body of work that has investigated lysosomal escape strategies for better targeting of other organelles. For example, Chen et al. demonstrated endosomal escape of DNA nanoribbons due to rigidity and high aspect ratio.^[^ ^73^ ^]^ The intracellular fate of DNA‐NPs is relatively better understood. Wu et al. demonstrated that DNA‐NPs can traffic through the endocytic pathway into late endosomes, residing there for up to 24 h following incubation. Notably, the DNA‐NPs were not observed to enter the lysosomes. After assessing the fate of the NP core and DNA shell, the authors showed that the oligonucleotide shell of the DNA‐NP was recycled out of the cell, while the NP cores remained inside.^[^ ^74^ ^]^

### 2.3 DNA‐NS‐Based Theranostics

Theranostic approaches combine diagnostics with a therapeutic approach for the treatment of cancer.^[^ ^75^ ^]^ The aim is to eliminate unnecessary invasive procedures such as biopsies and reducing delays in treatment, ultimately improving patient care and survival rates, while at the same time improving patient compliance by simplifying the clinical workflow. For example, DNA aptamers provide a means of selectively recognizing, targeting specific cells and also can act as a drug delivery platform. Thus, owing to its high stability, controllable, and cost‐efficient synthesis, DNA can be used as a scaffold to engineer DNA‐NSs for numerous biomedical applications, including those with a theranostic approach.^[^ ^75^, ^76^ ^]^

Shu et al. constructed multifunctional RNA NPs using the three‐way junction motif, thus manipulating the properties of RNA for targeted imaging and therapy of triple negative breast cancer.^[^ ^77^ ^]^ To precisely guide the anti‐miRNAs to the cancerous cells, epidermal growth factor receptor (EGFR) aptamers were used as targeting ligands to internalize the RNA NPs into cancer cells via receptor‐mediated endocytosis and then imaged using the fluorescent agent, Alexa674. The DNA‐NSs demonstrated high RNase resistance and thermodynamic stability and in addition, remained intact after systemic injection. Strong binding and internalization into cancerous tissue with little or no accumulation in healthy organs 8 h after administration were also observed. The authors noted the potential of incorporating drug molecules into such nanostructures for clinical applications involving cancer therapy.

**Figure 6:**
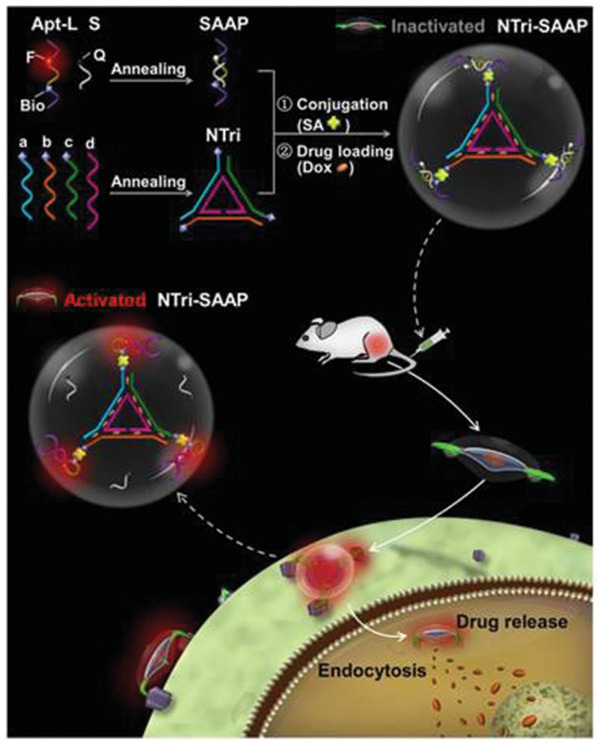
Schematic and theranostic activity of the DNA nanotriangle structure. By conjugating the DNA nanotriangle scaffold and multiple split activable aptamer probes (SAAPs), via a–c) three exterior strands and d) an inner strand, the DNA nanostructure is formed. In the free state, the DNA nanotriangle, which is loaded with DOX via the CG base pair regions is a flat, double helix‐jointed structure with no fluorescence since it is quenched. Once it encounters the target cell, the aptamers disassemble and consequentially change shape. This leads to fluorescence emission and partial drug release with the remaining drugs being freed following internalization. Reproduced under the terms of the Creative Commons Attribution License.^[^ ^80^ ^]^ Copyright 2018, Ivyspring International Publisher.

Doxorubicin (DOX) is a well‐established anticancer agent that disrupts the replication of cancer cells through DNA intercalation.^[^ ^78^ ^]^ QD655‐labeled triangular DNA origami structures loaded with DOX were administered to tumor‐bearing mice and, using noninvasive fluorescence imaging, tumor growth in mice and antitumor efficacy of drug‐loaded DNA carriers was dynamically monitored. The results demonstrated that the DNA origami dual imaging and drug delivery system showed optimal anticancer efficacy in vivo without observable systemic toxicity.^[^ ^79^ ^]^ Moreover, it was shown that triangular DNA‐NSs remained in the tumor for a longer period compared to square and tubular structures. The authors attributed the enhanced tumor retention times to the shape of the NS. Further, the DNA‐NSs offered additional layers of tightly packed double helices for DOX binding, allowing a higher payload of DOX to be delivered. As a consequence of their compact structure, origami nanostructures can be loaded with high drug concentrations, allowing them to be digested slowly in vitro and in vivo, therefore reducing unintentional drug release during trafficking of the DNA‐NSs to the tumor site in vivo.^[^ ^79^ ^]^ Lei et al. recently used a DOX‐loaded DNA nanotriangle‐scaffold aptamer probe for cancer detection, imaging, and therapy in both in vitro and in vivo settings. Superior targeted binding affinity with an ultralow nonspecific background was observed, thus generating high contrast tumor imaging within an extended time window of 8 h. Moreover, as a consequence of a five‐time improvement in DOX loading capabilities in comparison to the control, the DNA nanotriangle‐scaffold aptamer demonstrated high antitumor efficacy in vivo with 81.95% inhibition and no observable bodyweight loss, therefore indicating favorable biocompatibility (**Figure** **6**).^[^ ^80^ ^]^ DNA dendrimers have also been developed as dual imaging and drug delivery platform. The nanostructures were assembled from three‐armed Y‐shaped DNA monomers using an enzyme‐free step‐by‐step base‐pairing assembly strategy. The resulting structure served as a mechanism for cancer imaging and drug delivery through the incorporation of fluorophores, targeting DNA aptamers and DOX. The structure displayed a high degree of biostability and biocompatibility as well as high selectivity and high drug loading capacity, therefore making it a propitious candidate for applications involving DNA‐based theranostics.^[^ ^81^ ^]^

DNA nanorobots conjugated with dual functioning nucleolin targeting aptamers have been investigated for the delivery of thrombin. Such nanostructures are capable of selectively targeting the tumor environment, triggering the DNA nanorobot to open and release the therapeutic payload. Following the release of thrombin to the tumor site, vascular occlusion of the tumor vessels was observed, which in turn induced blockage of tumor blood supply, and inhibition of tumor growth to a high degree. In vivo imaging demonstrated specific tumor targeting with high‐intensity fluorescence signal observed in the tumor region 8 h post‐injection. Furthermore, in comparison to the controls, tumor volume was significantly smaller following treatment with the targeted thrombin nanotube, and the delivery of the DNA‐thrombin nanorobot prevented metastasis, thus showing excellent therapeutic potential.^[^ ^82^ ^]^ Inspired by the “envelope” of viral particles, Perrault and Shih encased a DNA octahedron into a PEGylated lipid bilayer to mimic virus‐like particles.^[^ ^64^ ^]^ The wireframe DNA nanooctahedron with a diameter of ≈50 nm was composed of bundles of six long double helices engineered with 90° curvatures, allowing the lipid bilayer to be directly assembled around the DNA octahedron. The authors demonstrated the envelopment of DNA nanostructures into PEGylated lipids, which were successfully protected against nuclease digestion. Activation of the immune system was decreased by two orders of magnitude below the controls, and pharmacokinetic bioavailability was enhanced significantly by a factor of 17. Therefore, the authors demonstrate the development of sophisticated, translation‐ready DNA nanodevices capable of being functionalized by targeting moieties to specifically target cancer tissue in vivo. Using a transdermal drug delivery approach, Wiraja et al. utilized DNA origami nanostructures to deliver DOX for the treatment of melanoma in vivo.^[^ ^83^ ^]^ Specifically, the DOX‐loaded DNA‐NS generated more than two‐fold improvement in drug accumulation and tumor growth inhibition compared to topically applied free DOX or DOX loaded in liposomes and polymeric NPs. In vivo fluorescence imaging was used to demonstrate successful skin penetration of the DNA‐NS to the dermis.

Several cancer types are associated with a mutated p53 gene, which is important in tumor suppression.^[^ ^84^ ^]^ Research suggests that p53 gene expression can enhance the sensitivity of multidrug‐resistant (MDR) tumors to chemotherapeutics. More recently, Ding et al. designed a DNA‐based co‐delivery system which contained a linear tumor therapeutic gene (p53) and DOX for the combined therapy of MDR tumor (MCF‐7R) using a MUC1 aptamer‐mediated targeted delivery.^[^ ^85^ ^]^ The structure, which resembles a kite (“nanokite”), exhibited superior anticancer activity against MCF‐7R both in vitro and in vivo. Fluorescence imaging of the Cy5.5‐labeled DNA nanokite structure also indicated preferential uptake of the targeted MUC1 aptamer in tumors over the nontargeted group. Compared to the control, the nanocarrier was also shown to inhibit tumor growth in vivo, demonstrating an almost 20‐fold higher level of p53 expression in tumors treated with the dual‐therapeutic p53 and DOX‐loaded DNA‐NS.

Owing to its biocompatibility, several studies have also explored the use of DNA nanostructures as a carrier for photosensitizers. Photodynamic therapy (PDT) is a promising technique that utilizes the sensitivity of a drug to a specific wavelength of light to create reactive oxygen species (ROS), killing the cancerous cells. In addition to directly killing cancer cells, PDT can shrink or destroy tumors in two other ways; photosensitizer induced damage of tumor vasculature and activation of the immune system to attack tumor cells.^[^ ^86^ ^]^ Targeted PDT was demonstrated by Tan et al. using aptamers to recognize cancerous cells selectively.^[^ ^87^ ^]^ Toehold‐mediated catalytic strand displacement caused by the overhanging catalyst sequence on the aptamer resulted in the activation of the photosensitizer molecule chlorin e6 (Ce6), which, in turn, enhanced the therapeutic effect. Importantly, through the use of a targeting sequence, catalytic amplification of Ce6 took place close to the cancerous cells, thus amplifying the local concentration of singlet oxygen. Similarly, a DNA‐based nanoclaw has been designed for targeted cancer therapy using PDT.^[^ ^88^ ^]^ Consisting of an oligonucleotide backbone as the scaffold, Ce6 was incorporated into the aptamer‐based nanostructure which was capable not only of selectively targeting cancerous cells but also administering a diagnostic signal such as fluorescence as well as PDT. The results demonstrate the advantage of using logic‐based aptamer nanostructures for cancer detection and treatment applications. Although PDT is an FDA (The Food and Drug Administration)‐approved method of treatment for certain cancers, its application remains limited due to the poor penetration depth of UV and visible light in tissue. To account for this, Liang et al. utilized a DNA origami nanostructure for dual functioning imaging and PDT applications using the carbazole derivative 3,6‐bis[2‐(1‐methylpyridinium)ethynyl]‐9‐pentyl‐carbazole diiodide (BMEPC).^[^ ^89^ ^]^ The molecule exhibits low solubility in aqueous samples. However, the advantage of using such a molecule is its ability to be activated by near‐infrared (NIR) light which has better tissue penetration capabilities. Using DNA as a carrier to overcome the issues associated with solubility and quenching of fluorescence due to aggregation, DNA was used as a carrier to increase the density of BMEPC molecules in the cellular environment. Through the use of BMEPC as a photosensitizer and imaging probe, increased fluorescence emission and higher cell apoptosis were observed as a consequence of free radical production.

**Figure 7:**
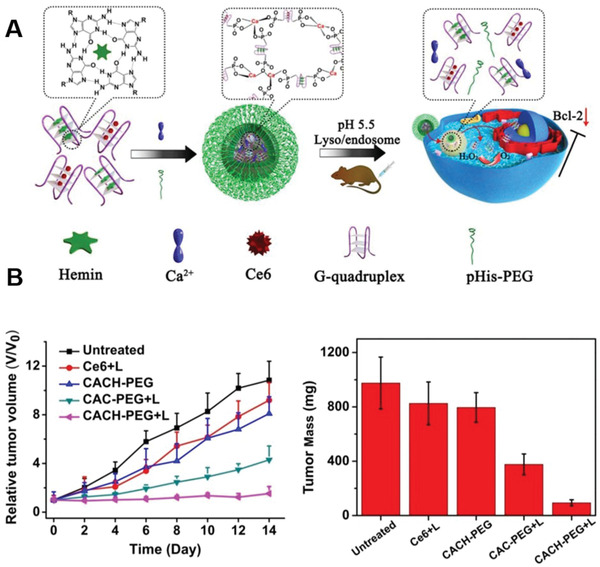
Schematic representation of the synthesis of CACH‐PEG and its effects in vivo. a) Hemin and Ce6 are inserted into the G‐quadruplex structure. Mixing of the G‐quadruplex structure and Ca^2+^ together with pHis‐PEG yields Ca‐AS1411/Ce6/hemin@pHis‐PEG(CACH‐PEG) NCPs, which are pH responsive at ≈5.5 and were reduced to smaller G‐quadruplex complexes in acidic environments of the lyso‐ and endosomes. b) The tumor growth curves of 4T1‐tumor‐bearing mice following each treatment as indicated. Tumors were irradiated using 660 nm light‐emitting diode light (5 mW cm^−2^) for 60 min. c) Average tumor weights collected from the each group following 14 days after each treatment. Reproduced with permission.^[^ ^90^ ^]^ Copyright 2018, American Chemical Society.

By utilizing coordination chemistry to induce self‐assembly, Chen et al. created novel DNA nanoscale coordination polymers (NCPs).^[^ ^90^ ^]^ DNA NCPs were created by mixing Ca^2+^, AS1411 G quadruplex, and poly His‐PEG copolymer (**Figure** **7**a). In addition, photosensitive Ce6 and hemin were inserted into the DNA‐NS to enable targeted intranuclear delivery of Ce6 which, following exposure to 660 nm light, induce the generation of ROS inside the cell nuclei. Tumor growth inhibition was observed to be most effective in groups treated in this manner compared to all other control groups (Figure 7b,c). Efficient tumor accumulation was demonstrated in vivo using fluorescence and single photon emission computed tomography (SPECT) imaging, supported by radiolabeling Ce6 within the NCPs with ^99m^Tc^4+^ ions. Furthermore, the inhibition of antiapoptotic protein B‐cell lymphoma 2 (Bcl‐2) expression by AS1411 supported PDT‐induced cell apoptosis. This work offers an efficient means of achieving intranuclear delivery of photosensitizers and down‐regulation of antiapoptotic proteins for applications involving cancer theranostics.

**Figure 8:**
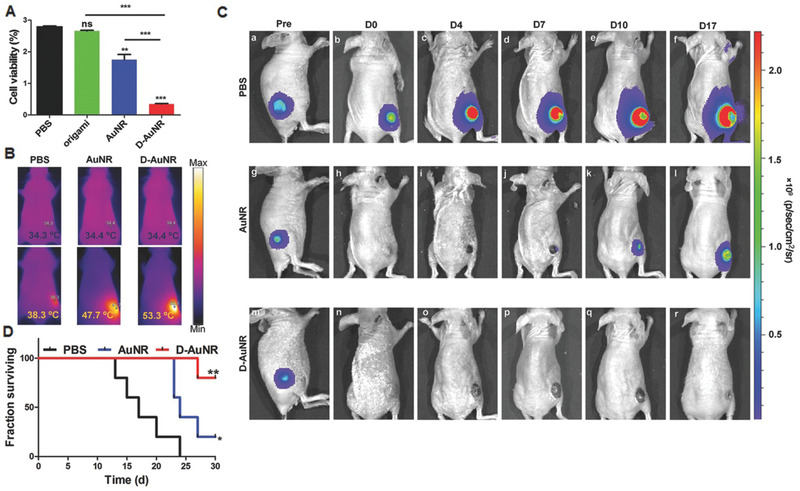
Demonstration of the efficiency of photothermal therapy using either AuNR or DOX–AuNR. a) Cell viability of 4T1‐fLuc tumor cells following administration of varying treatments followed by NIR laser irradiation (808 nm, 1.5 W cm^−2^, 3 min) (**p < 0.01, ***p < 0.0001). b) IR thermographic maps of each group of mice obtained 10 min after NIR irradiation. c) Bioluminescence imaging (BLI) of 4T1‐fLuc‐tumor‐bearing mice intravenously injected with PBS (control), AuNR and D–AuNR in combination with NIR laser irradiation acquired prior to injection and at days 0, 4, 7, 10, and 17. d) Survival rates of each group of mice following treatment were monitored 30 days post‐injection. Reproduced with permission.^[^ ^91^ ^]^ Copyright 2016, WILEY‐VCH.

**Figure 9:**
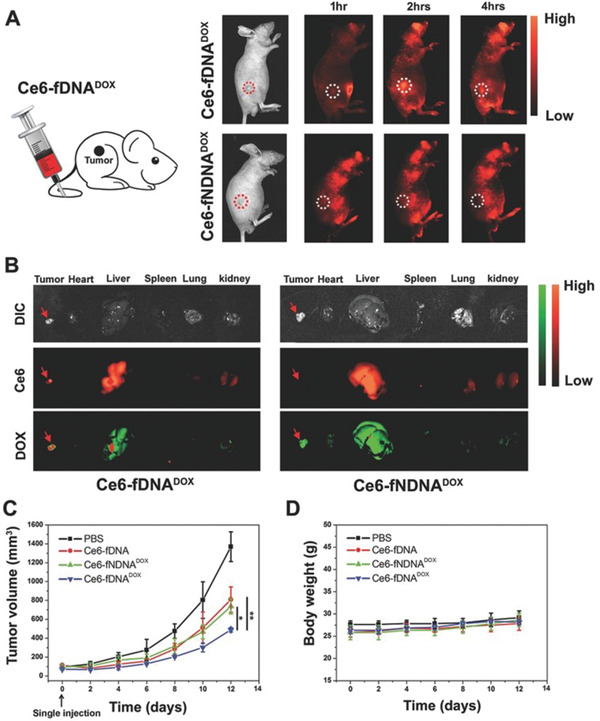
a) Fluorescence imaging of HepG2 tumor‐bearing mice injected with either Ce6‐fDNADOX or Ce6‐fNDNADOX. Time lapse fluorescence images of Ce6 in vivo confirm tumor uptake. b) White light and fluorescence images of different organs removed from HepG2 tumor‐bearing mice 2 h post‐injection of either Ce6‐fDNADOX or Ce6‐fNDNADOX where Ce6 is represented in red and DOX in green. c) Tumor volume of mice (*n =* 5) after single IV injection of the indicated treatments. Tumors were imaged 2 h after injection by irradiation with 670 nm light (0.2 W cm^−2^) for 10 min. d) Average body weight of mice following each treatment (*n* = 5). Reproduced under the terms of the Creative Commons Attribution License.^[^ ^93^ ^]^ Copyright 2017, WILEY‐VCH.

DNA‐NPs have also shown promise in overcoming the limitations associated with the lack of effective contrast agents in photoacoustic imaging.^[^ ^91^, ^92^ ^]^ By assembling gold nanorods (AuNRs) onto DNA origami structures, a DNA‐based optoacoustic imaging system was reported by Du et al.^[^ ^91^ ^]^ The authors showed that the resulting AuNR‐based DNA‐NS hybrid demonstrated exceptional properties, serving as both a unique probe and an efficient contrast agent for in vivo optoacoustic imaging by generating significant improvements in image quality and contrast. Simultaneous photothermal therapy (PTT) was achieved as a response to NIR irradiation and thus inhibited the regrowth of tumors and prolonged the survival rate of mice bearing 4T1‐tumors (**Figure** **8**). In response to the TME, Zhang et al. designed a self‐assembling Ce6‐ fDNA‐DOX hybrid capable of performing cancer cell‐specific fluorescence imaging. Synergistic redox‐responsive PDT and pH‐triggered chemotherapy was also achieved through the release of DOX in hepatocellular carcinoma.^[^ ^93^ ^]^ Here, the smart Ce6‐fDNA‐DOX therapeutic NS overcame unwanted side effects by switching the PDT “on” and “off” as necessary. Treatment outcomes were also improved due to the synergistic use of DOX. Fluorescence emission of DOX from Ce6‐fDNA‐DOX was significantly greater, in comparison to the nontargeted control (Ce6‐fNDNA‐DOX) (**Figure** **9**a,b), indicating that smart DNA‐NSs can be better incorporated into HepG2 cells due to specific targeting. In comparison to the control group (phosphate‐buffered saline, PBS), injection of Ce6‐fDNA, Ce6‐fNDNADOX, or Ce6‐fDNADOX followed by 670 nm irradiation demonstrated noticeable delay of tumor growth (Figure 9c). Importantly, mice that were administered the dual therapy, i.e., the Ce6‐fDNADOX treatment in combination with PDT (670 nm), exhibited significantly slower tumor growth due to the synergistic therapeutic effects of PDT and chemotherapy. Also, no discernable weight loss was observed, indicating low toxicity of the Ce6‐fDNADOX probe (Figure 9d). Other DNA‐NSs responsive to the pH of the tumor environment include one reported by Di et al. for the controlled imaging of adenosine triphosphate (ATP) in the extracellular tumor matrix.^[^ ^94^ ^]^ As a consequence of mild acidity (lower pH), specific anchoring of aptamer units to the membrane of tumor cells induced off–on fluorescence, thus enabling fluorescence imaging of extracellular ATP with high signal to background ratios in solid tumors. Relevant references with in vivo studies are summarized in **Table** **1**.

**Table 1:** Summary of DNA‐NS‐based theranostics and in vivo applications

Reference number	Type of NS	Imaging modality	Applications
^[^ ^77^ ^]^	RNA nanostructure	Fluorescence	Delivery of miRNA‐based therapeutics
^[^ ^79^ ^]^	DNA origami triangle	Fluorescence	Imaging and therapy of breast cancer
^[^ ^80^ ^]^	DNA nanotriangle	Fluorescence	Bioimaging and drug delivery of DOX
^[^ ^64^ ^]^	DNA octahedron	Fluorescence	Assessment of immune activation
^[^ ^83^ ^]^	Different 2D and 3D origami structure	Fluorescence	Transdermal delivery of DOX
^[^ ^85^ ^]^	DNA origami triangle	Fluorescence	Combined therapy for MDR‐resistant breast cancer
^[^ ^90^ ^]^	DNA nanogel	Fluorescence and PET	Intranuclear delivery of photosensitizers, down‐regulation of antiapoptotic proteins, and modulation of an unfavorable TME
^[^ ^91^, ^92^ ^]^	DNA origami triangle‐AuNR hybrid	Optoacoustic imaging	Improved optoacoustic and excellent photothermal therapeutic properties
^[^ ^93^ ^]^	Double stranded DNA hybrid	Fluorescence	Redox‐responsive PDT and pH‐triggered DOX release
^[^ ^94^ ^]^	Streptavidin DNA hybrid nanomachine	Fluorescence	Controlled imaging of ATP

### 2.4 DNA‐NP‐Based Theranostics

In this emerging field, many studies have aimed at understanding simultaneous transfection and gene regulation using DNA‐NPs. In contrast with conventional approaches to gene delivery, DNA‐NPs do not require cationic transfection agents or additional structural modifications to aid cellular entry.^[^ ^95^ ^]^ In 2006, Rosi et al. first demonstrated that DNA‐NPs could deliver “antisense” oligonucleotides to eukaryotic cells combined with a tuneable enhanced green fluorescence protein (EGFP) knockdown.^[^ ^70^ ^]^ Later, Giljohann et al. reported the successful delivery of siRNA molecules using a polyvalent RNA‐gold NP (RNA‐Au NPs) in a human cancer cell line.^[^ ^67a^ ^]^ The RNA‐Au NPs exhibited a greater half‐life than free dsRNA, were capable of entering cells without the use of transfection agents, and exhibited high gene knockdown capabilities in vitro. There are many more examples of in vitro gene silencing reviewed elsewhere.^[^ ^28^ ^]^

**Figure 10:**
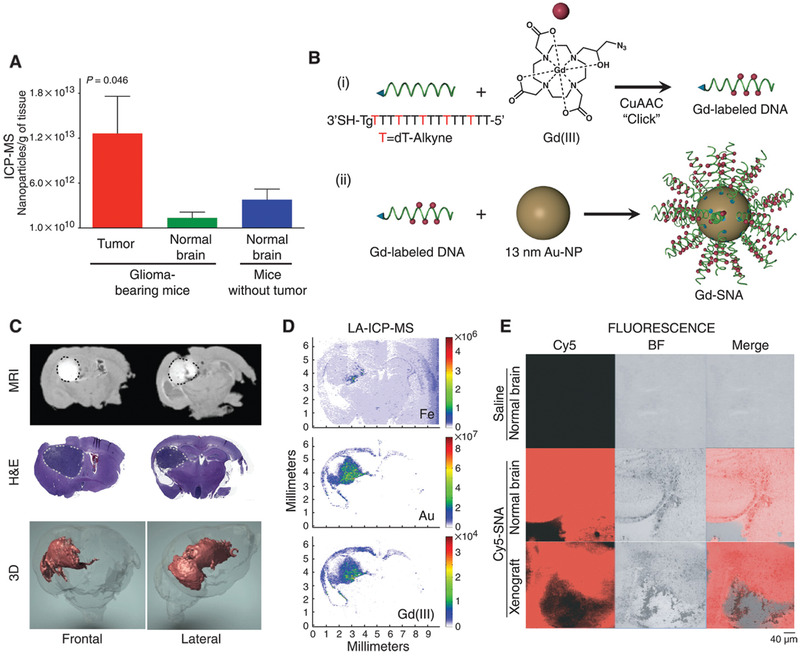
DNA‐NPs distributed throughout glioma tissue. a) Quantification of DNA‐NP uptake into orthotopic U87MG tumor and adjacent normal tissue at 48 h post‐injection using inductively coupled plasma mass spectrometry (ICP‐MS). b) Schematic of the synthesis of Gd(III)‐functionalized DNA‐NPs. c) Accumulation of Gd(III)‐DNA‐NPs within the intracerebral lesion is confirmed using magnetic resonance imaging (MRI), hematoxylin and eosin staining, and 3D reconstructions. d) Localization of Au, Fe, and Gd(III) contents in coronal brain sections of mice injected intracranially with Gd(III)‐DNA‐NPs as confirmed by LA‐ICP‐MS. e) Confocal fluorescence microscopy of coronal brain sections derived from tumor‐ and nontumor‐bearing mice following saline or Cy5‐DNA‐NP injection. Reproduced with permission.^[^ ^96a^ ^]^ Copyright 2013, American Association for the Advancement of Science.

DNA‐NPs have been utilized for the targeting of several genes such as Bcl2L12, miR‐182, ganglioside GM3 synthase, EGFR, Malat‐1 in vivo.^[^ ^96^ ^]^ Jensen et al. evaluated an RNA interference (RNAi)‐based nanotheranostic for the neutralization of oncogene expression in glioblastoma multiforme (GBM). DNA‐NPs consisted of AuNPs grafted with densely packed and highly oriented si‐RNA duplexes. Excitingly, the NPs crossed the blood–brain barrier (BBB) to accumulate throughout the tumor mass in GBM mouse models (**Figure** **10**).^[^ ^96a^ ^]^ The NPs were designed to target the oncoprotein Bcl2Like12 (Bcl2L12), an effector caspase, and p53 inhibitor overexpressed in GBM relative to a healthy brain. An efficient knockdown of endogenous Bcl2L12 mRNA and protein levels was observed, and glioma cells underwent therapy‐induced apoptosis by enhancing effector caspase and p53 activity. Later, using a similar approach, NPs were developed to deliver siRNA and miRNA to intracranial GBM tumor sites.^[^ ^97^ ^]^ A reporter xenograft model that was able to stably co‐express optical reporters for luciferase and an NIR fluorescent protein (iRFP670) was developed to evaluate efficacy in vivo. Using noninvasive optical imaging, knockdown of the DNA repair protein O6‐methylguanine‐DNA‐methyltransferase (MGMT, linked to drug resistance in GBM), by NPs composed of MGMT‐targeting siRNA duplexes was quantitatively assessed. A systemic administration of NPs via single tail vein injection was shown to be capable of robust intratumoral MGMT protein knockdown. Further, NP biodistribution and pharmacokinetics revealed rapid intratumoral uptake and retention, therefore improving the antitumor activity of co‐administered temozolomide (TMZ). These NPs exhibited no observable toxicity, as corroborated by histopathology and blood chemistry assessment.

The topical application of DNA‐NPs offers potential therapeutic advantages for the treatment of skin diseases. Zheng et al. demonstrated that DNA‐NPs freely penetrate keratinocytes in vitro (e.g., mouse skin and human epidermal tissue) within hours of post‐application despite the fact that the intact epidermal barrier typically precludes entry of gene‐suppressing therapy. Significantly, the NPs were delivered in a commercially available moisturizer or PBS and importantly, no barrier‐disrupting or transfection agents, such as liposomes, peptides, or viruses were required. NPs were designed to target EGFR and were >100‐fold more potent than siRNA delivered with commercial lipid agents in vitro. Furthermore, they completely abolished EGFR expression, suppressing downstream ERK phosphorylation, and reduced epidermal thickness by almost 40%. As such, DNA‐NPs were shown to be very promising agents for topical gene therapy of neoplastic, inflammatory, and genetic disorders of the skin.^[^ ^98^ ^]^

**Figure 11:**
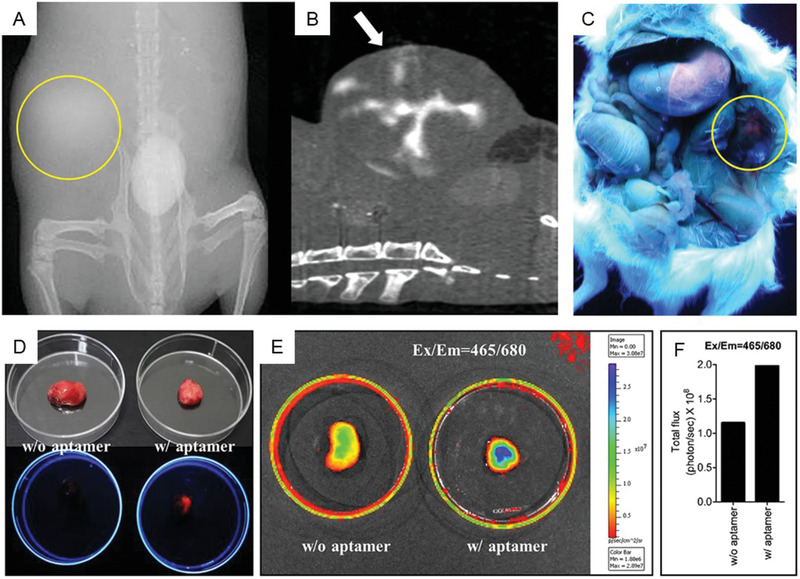
a–c) X‐ray image, axial CT image, and fluorescence image of the CL1‐5 tumor‐bearing mouse taken 30 min post‐injection of aptamer‐targeted AuNPs as indicated by the yellow circle, white arrow, and yellow circle, respectively. d) CL1‐5 tumors under white light (top) and UV light (bottom). e) CL1‐5 tumors incubated without and with aptamers. f) Calculated total photon fluxes. Reproduced under the terms of the Creative Commons Attribution License.^[^ ^103^ ^]^ Copyright 2015, Springer Nature.

**Figure 12:**
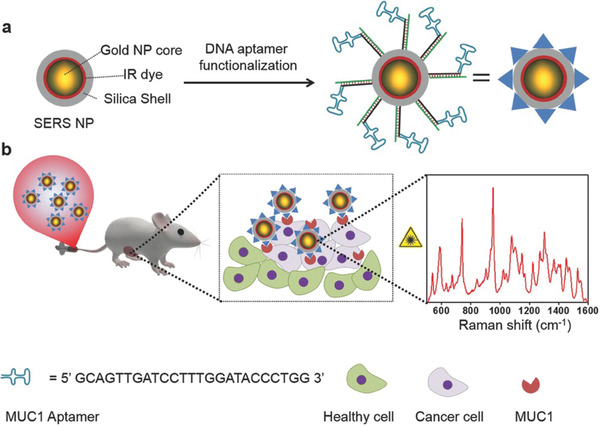
a) Schematic illustration of MUC1‐functionalized SERS NPs. b) The MUC1 aptamer‐functionalized DNA‐NPs were administered intravenously to human breast cancer tumor‐bearing mice and selectively targeted the tumors over‐expressing MUC1. Their accumulation within tissue was confirmed via identification of the “fingerprint” spectral signature of the SERS NPs using Raman spectroscopy. Reproduced with permission.^[^ ^106^ ^]^ Copyright 2017, WILEY‐VCH.

Recently, aptamers, specific sequences of DNA or RNA molecule selected for binding specific targets, have been shown to be promising platforms for targeting cancer biomarkers with high selectivity and specificity.^[^ ^99^ ^]^ Aptamers have multiple benefits over monoclonal antibodies such as i) lower molecular weight, ii) low immunogenicity, iii) inexpensive synthesis via solid‐phase phosphoramidite chemistry, and iv) available chemical modifications for attachment to the NP surface. Specifically, gold nanomaterials have been extensively investigated as core materials for aptamer‐targeted theranostic applications.^[^ ^100^ ^]^ Aptamer DNA‐NPs have been used in both in vitro and in vivo applications. Nicholls et al. reported a gold NP functionalized with altered deoxythymidine oligonucleotides bearing Gd(III) chelates and a fluorescent Cy3 moiety to enable the visualization of transplanted human neural stem cells in vivo. The DNA‐NPs exhibited improved T1 relaxivity and excellent cellular uptake. In vivo, MRIs were corroborated with histological studies. The DNA‐NPs thus offer new opportunities of visualizing transplanted cells using MRI as a tool to derive biologically relevant information.^[^ ^101^ ^]^ Melancon et al. developed aptamer‐coated hollow gold NPs targeted to EGFRs. EGFR‐targeting RNA aptamers were attached to a thiol‐terminated single‐stranded DNA grafted to hollow gold NPs. The pharmacokinetics, biodistribution, and micro‐SPECT/CT imaging of ^111^In‐labeled targeted DNA‐NPs were evaluated in vivo in oral cancer mouse models. Micro‐SPECT/CT imaging further confirmed tumor homing of DNA‐NPs.^[^ ^102^ ^]^ Later, Li et al. demonstrated the fabrication of a fluorescent and CT dual‐modal DNA‐NP, conjugated with diatrizoic acid and nucleolin‐targeted aptamers. The fluorescent NP conjugates were utilized as a molecular contrast agent to thus reveal the tumor location in the human lung adenocarcinoma cell line (CL1‐5) in tumor‐bearing mice by CT imaging. Furthermore, the orange‐red fluorescence emitted from the conjugates in the lung adenocarcinoma could be visualized by the naked eye (**Figure** **11**). Following tumor resection, the tumor's fluorescence signal was significantly enhanced compared to that of the control as a result of tumor‐specific accumulation of the DNA‐NPs.^[^ ^103^ ^]^ Shi et al. designed a theranostic nanoprobe by conjugating aptamer probes onto Au@Ag/Au NPs and utilizing them in image‐guided cancer therapy of lung cancer mouse models. In this study, The Au@Ag/Au NPs with a large absorption cross‐section from 400 to 1100 nm functioned as the fluorescence quencher and optical heater with a higher capacity for hyperthermia compared to AuNRs. By incorporating a target‐specific signal alteration mechanism, the DNA‐NPs substantially improved the imaging contrast with a shortened detection time and improved PTT potency.^[^ ^104^ ^]^ Ye et al. reported aptamer‐functionalized Cu‐Au alloy nanostructures for in vivo cancer theranostics. Due to the excellent thermal conductivity and lower cost of copper (Cu), Cu‐Au alloy NPs were successfully synthesized in one‐pot synthesis method. The NPs demonstrated superior thermo‐optical properties, including a broad and intense NIR absorption band, and demonstrated superior heating performance using incident light of different wavelengths and stability against melting. By using a human leukemia CCRF‐CEM cancer cell line, selective fluorescence imaging and NIR photothermal therapy were demonstrated using Cy5‐labeled aptamer‐coated Cu‐Au alloy NPs.^[^ ^105^ ^]^ Pal et al. developed a strategy to functionalize SERS NPs with a DNA aptamer to target Mucin1 (MUC1) in human breast cancer. MUC1‐targeted SERS NPs were co‐injected with nonspecific SERS NPs with different spectral signatures in a breast cancer xenograft mouse models. A two‐tumor mouse model with differential expression of MUC1 was used to demonstrate that the targeted SERS NPs accumulate in the TME via active targeting of MUC1 (**Figure** **12**).^[^ ^106^ ^]^ Recently, Pal et al. also reported a DNA‐based strategy for multimodal NPs with complementary fluorescence and SERS modalities for cancer imaging and therapy using mouse models of ovarian and glioblastoma.^[^ ^107^ ^]^ This year, Di et al. reported an up‐conversion DNA‐NP‐based nanodevice which could be activated using two independent NIR lights for programmable activation of aptamer‐targeting modules and a photosensitizer. The authors showed excellent spatiotemporal target activation and photodynamic antitumor effect in vivo.^[^ ^108^ ^]^ Relevant references with in vivo studies are summarized in **Table** **2**.

**Table 2:** Summary of DNA‐NP‐based theranostics and in vivo applications

Reference number	Type of NP core	Imaging modality	Applications
^[^ ^96a^ ^]^	13 nm spherical AuNP	MRI, fluorescence, ICP‐MS (ex vivo)	RNAi therapy for GBM
^[^ ^97^ ^]^	13 nm spherical AuNP	Indirect imaging with fluorescence and bioluminescence	Real‐time assessment of MGMT‐targeting spherical nucleic acids
^[^ ^98^ ^]^	13 nm spherical AuNP	Fluorescence	EGFR siRNA‐based gene silencing
^[^ ^101^ ^]^	13 nm spherical AuNP	Fluorescence and MRI	To detect transplanted human stem cell
^[^ ^102^ ^]^	Hollow gold nanospheres	*μ*SPECT‐CT	Targeted imaging of EGFR positive head and neck cancer
^[^ ^103^ ^]^	2.4 nm AuNP	CT, fluorescence	Nucleolin targeted imaging of lung cancer
^[^ ^104^ ^]^	Au@Au/Ag NP	Activatable fluorescence	Imaging and PTT of lung cancer
^[^ ^105^ ^]^	Cu‐Au alloy nanostructure	Fluorescence	Targeted imaging and therapy of leukemia
^[^ ^106^ ^]^	60 nm AuNP with silica	Raman Imaging	Targeted imaging of breast cancer
^[^ ^107^ ^]^	AuNR with silver and silica shell	Raman and fluorescence imaging	Targeted imaging and PTT of ovarian cancer and GBM
^[^ ^108^ ^]^	Lanthanum‐doped up‐conversion nanoparticle with silica shell	Fluorescence	Targeted photodynamic therapy of breast cancer

### 2.5 DNA‐NSs as Drug and Gene Delivery Platforms

It is clear that important advancements have been made in developing effective DNA‐NS and DNA‐NPs for theranostic applications. However, it is also imperative to highlight recent progress that has investigated the use of these nanomaterials exclusively in drug and gene delivery applications. To be considered as an efficient drug and gene delivery system, both DNA‐NSs and DNA‐NPs must be designed to deliver the following features: i) structural stability in physiological conditions, ii) predictable and well‐defined structures, iii) high loading capacity and the ability to be internalized by cells, and iv) excellent biocompatibility.^[^ ^109^ ^]^ DNA‐NSs offer high loading capacity and can, therefore, be functionalized with a range of molecules, including pharmaceuticals, e.g., DOX, by means including covalent modification or intercalation.^[^ ^109^, ^110^ ^]^ In addition, alongside their excellent biocompatibility and low toxicity, DNA‐NSs can be internalized into cancerous cells.^[^ ^111^ ^]^ As a consequence of these properties, DNA‐NSs have emerged as promising candidates for drug and gene delivery platforms.

**Figure 13:**
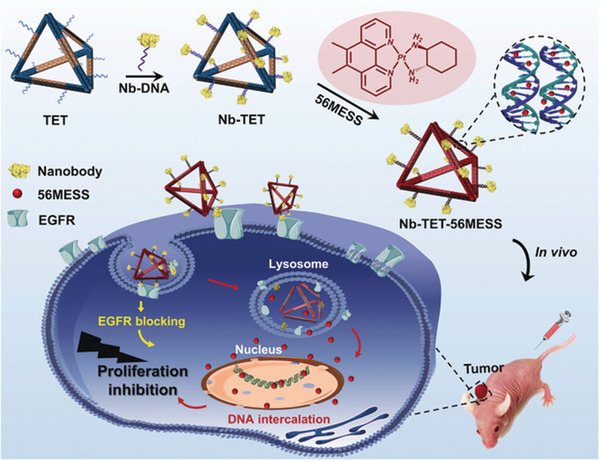
Conceptual figure illustrating the use of a nanobody (Nb)‐conjugated DNA‐NS for the delivery of the intercalating chemotherapeutic, 56MESS, where Nb, nanobody; TET, double‐bundle DNA tetrahedron; Nb‐TET, Nb‐conjugated double‐bundle DNA tetrahedron; Nb‐TET‐56MESS, platinum‐drug‐loaded NbTET; EGFR, epidermal growth factor receptor. Reproduced with permission.^[^ ^119^ ^]^ Copyright 2019, WILEY‐VCH.

Capable of suppressing gene expression, small interfering RNA (siRNA) has been investigated for disease therapy applications. However, due to the fragility of siRNA in plasma and reduced internalization without transfection agents, its therapeutic applications in vivo are limited.^[^ ^112^ ^]^ In spite of this, DNA‐NSs have been shown to be effective vehicles in the delivery of siRNA for cancer therapy. Lee et al. developed folic acid‐targeted self‐assembled tetrahedral DNA nanostructures (TDNs) for the delivery of siRNA into cells to silence target genes in tumors.^[^ ^113^ ^]^ Such TDNs demonstrated robust gene silencing via both intratumor and systemic injection, as well as boasting four times longer blood circulation time than free siRNA. In addition, controlled gene silencing was achieved only when the ligands were assembled in a suitable spatial orientation. Due to their structural rigidity and high aspect ratio, which makes them capable of escaping endosomal entrapment without involving a proton pump, Chen et al. explored the use of DNA nanoribbons as effective siRNA delivery vehicles for mediating gene silencing without the need for cationic transfection agents.^[^ ^73^ ^]^ The authors demonstrated the down‐regulation of surviving mRNA expression and protein production by 40.8% and 45.2%, respectively. As such, the capability of the DNA nanoribbons to escape from the endosomes opens up the possibility for the use as a delivery vehicle for siRNA and other biologics. Zhang et al. developed a novel siRNA delivery system in which siRNAs functioned as cross‐linkers to drive the self‐assembly of DNA‐grafted polycaprolactone brushes into spherical and nanosized hydrogels via nucleic acid hybridization. This ensured that the siRNAs were fully embedded and protected for systemic delivery to the site of interest.^[^ ^114^ ^]^ The siRNA‐embedded nanogels demonstrated resistance to RNase degradation, enhanced cellular uptake, accumulation, and consequential siRNA delivery to the tumor site, thus resulting in gene silencing in vivo. Bujold et al. constructed DNA “nanosuitcases” capable of encapsulating siRNA constructs and releasing them upon recognition of an oligonucleotide trigger such as mRNA or microRNA.^[^ ^115^ ^]^ Importantly, the DNA scaffold offered protection of the siRNA cargo from specific cleavage and nuclease degradation. Aptamer‐integrated DNA dendritic nanostructures have also been investigated for efficient siRNA delivery in which aptamers and siRNA were hybridized to the outermost layer of DNA dendritic nanostructures to enable the formation of aptamer–siRNA complexes. These complexes were investigated for the targeted delivery of siRNA in vitro gene therapy applications.^[^ ^116^ ^]^ DNA “Trojan horses,” self‐assembled from floxuridine‐containing DNA strands, have been utilized to deliver chemotherapeutics into cancer cells and tissues. The Trojan horse with buckyball architecture displays superior anticancer capability over free drugs.^[^ ^117^ ^]^ Zhang et al. conjugated camptothecin (CPT) molecules by directly reacting with phosphorothioate (PS)‐modified DNA to improve solubility. Further, the self‐assembled DNA‐NSs exhibited a decreased tumor growth rate in vivo.^[^ ^118^ ^]^ Recently, Wu et al. constructed an EGFR nanobody‐conjugated DNA‐NS‐based carrier of 56MESS, a platinum‐based intercalator for targeted delivery in vivo (**Figure** **13**).^[^ ^119^ ^]^ Interestingly, by exploiting the base‐pairing properties of DNA, the double‐bundle DNA tetrahedron was able to arrange anti‐EGFR nanobodies in a highly organized manner to achieve successful targeting. In addition, a DNA‐NS capable of delivering a vector of RNA interference (RNAi) and chemotherapeutic drug (DOX) for treatment of MDR tumors was reported by Liu et al.^[^ ^120^ ^]^ Owing to the high degree of arrangement associated with the DNA, two linear small hairpin RNA (shRNA) transcription templates were precisely organized in drug‐loaded DNA‐NS, to synergistically inhibit tumor growth in preclinical mouse models. Ma et al. reported an HER2 aptamer‐targeted DNA‐NS for lysosomal degradation of tumor‐specific protein molecules and apoptosis by selectively targeting HER2 positive breast cancer.^[^ ^121^ ^]^ This was achieved by anchoring anti‐HER2 aptamers onto the exterior DNA‐NS resulting in prolonged circulation, higher stability, and improved performance in vivo compared to the free anti‐HER2 aptamer.

**Figure 14:**
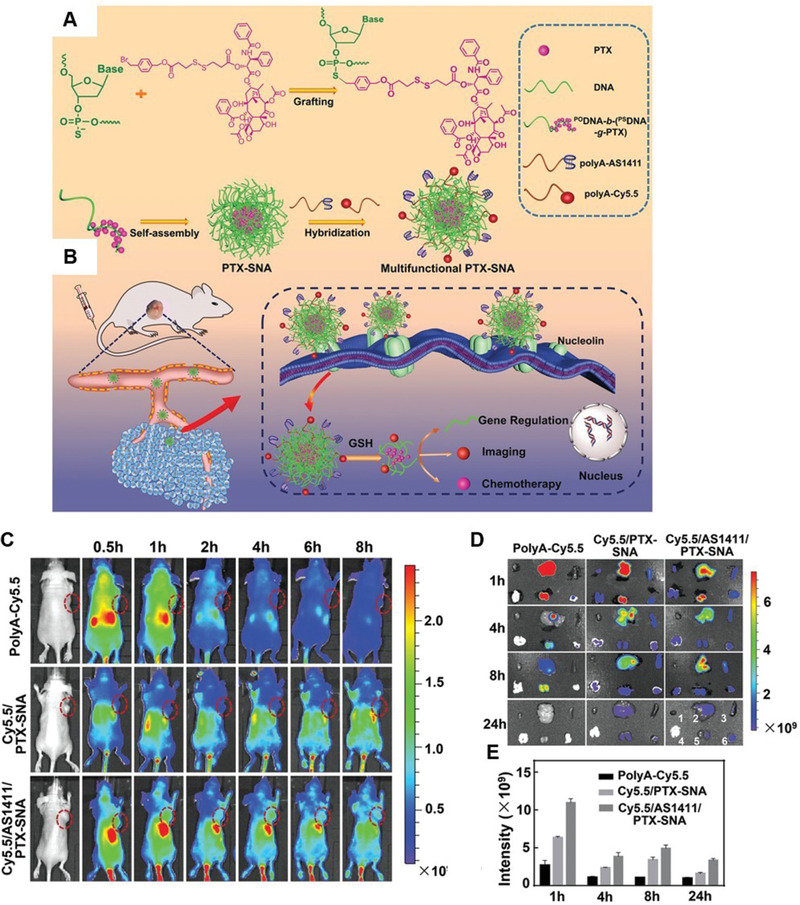
a,b) Schematic of the synthesis, self‐assembly, and theranostic potential of the DNA‐NSs for anticancer treatment. c,d) In vivo tumor targeting and biodistribution of each DNA‐NS formulation following administration. e) Semiquantitative analysis of fluorescence signals in tumors at 1, 4, 8, and 24 h time points. Reproduced with permission.^[^ ^124^ ^]^ Copyright 2019, WILEY‐VCH.

As well as being used as gene delivery vehicles, DNA‐NSs have also been investigated for the delivery of drug molecules, namely, intercalating drugs such as DOX.^[^ ^122^ ^]^ DNA‐NSs are particularly appealing vehicles for the delivery of such therapeutic molecules since they are capable of permeating the cell membrane, biocompatible, and can be adapted with targeting ligands such as aptamers. As such, TDNs have emerged as one of the most efficient DNA‐NSs for drug delivery applications, since they can be assembled from four ss‐DNA molecules and internalized without the need for transfection agents.^[^ ^123^ ^]^ TDNs were used for the delivery of paclitaxel (PTX) used in the treatment of cancers, including lung and ovarian. However, despite the pharmaceutical potency of PTX, its therapeutic value is limited by MDR. Lin et al. utilized TDNs for the successful delivery of PTX to nonsmall cell lung cancer cells and cells resistant to PTX. Importantly, the ability to override drug resistance and the downregulation of the MDR1 gene and P‐glycoprotein in PTX‐resistant cells was observed. TDNs functionalized with the aptamer AS1411 have also been utilized for targeting cells overexpressing nucleolin.^[^ ^72^ ^]^ A substantially higher number of aptamer‐targeted TDNs entered and accumulated in the nucleus of MCF‐7 cells compared to nontargeted TDNs, indicating the superiority of tumor‐targeted drug delivery TDNs over nontargeted TDNs. The authors envisage that such a platform could be utilized for the selective delivery of chemotherapeutic agents to target cells.^[^ ^72^ ^]^ Other DNA‐NSs for the delivery of small‐molecule chemotherapeutics include the use of di‐block DNA strands containing both normal phosphodiester segments (PODNA), and phosphorothioate segments (PSDNA) directly grafted with multiple PTX drug molecules, which then assemble into amphiphilic PTX‐­loaded spherical nucleic acid (SNA)‐like micellar NPs (PTX‐SNAs) (**Figure** **14**a,b).^[^ ^124^ ^]^ Significantly, multifunctional PTX­SNAs demonstrated high drug loading ratios (up to ≈53%) and achieved active‐targeting to inhibit tumor growth and reverse drug resistance in both in vitro and in vivo models. (Figure 14c–e).

**Figure 15:**
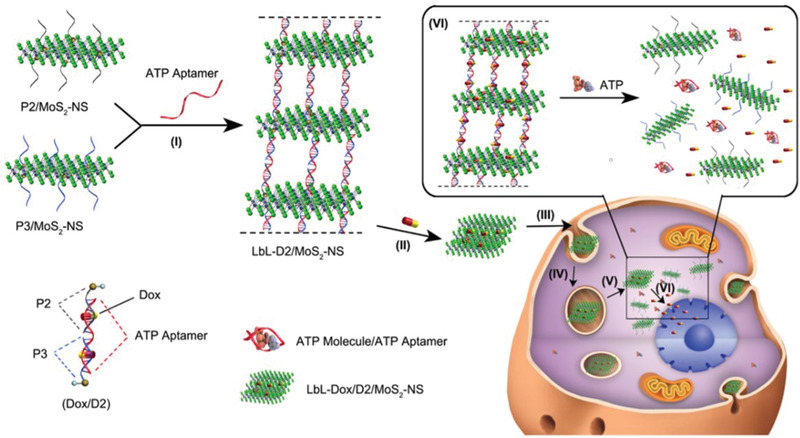
Schematic illustration of the formation of multilayer Dox/D2/MoS2‐NS and subsequent intracellular release of DOX in which ATP‐aptamers enable layer‐by‐layer assembly. DOX is loaded within the multilayer structure. Cellular uptake is achieved through endocytosis and ATP‐induced DOX release occurs in the cytosol. Reproduced with permission.^[^ ^127^ ^]^ Copyright 2017, American Chemical Society.

Zhao et al. tuned DNA origami nanostructures for the optimal delivery of DOX to human breast cancer cells. To rationally control and tailor drug release kinetics, the DNA‐NSs were designed to exhibit varying degrees of global twist, which resulted in different amounts of relaxation in the double helix structure. In addition to controlling the degree of drug encapsulation and release rate, increased cytotoxicity and lowered intracellular elimination were reported in contrast to free DOX.^[^ ^125^ ^]^ Similarly, Halley et al. observed increased delivery and retention of daunorubicin, which, similar to DOX, is capable of intercalation. Rod‐like DNA origami was used as a vehicle for the delivery of daunorubicin to a leukemia cell line exhibiting MDR.^[^ ^126^ ^]^ In addition to DNA origami structures, ultrathin 2D nanosheets of layered transition metal dichalcogenides, e.g., MoS_2,_ have attracted increasing attention recently due to their suitability in a range of applications, including biomedicine (**Figure** **15**).^[^ ^127^ ^]^ By taking the advantage of sulfur atom defect vacancies on MoS_2_, Li et al. functionalized otherwise inert MoS_2_ nanosheets with sulfur‐terminated DNA oligonucleotides. DOX was loaded into the DNA/MoS_2_ nanosheet structure and was protected from DNA intracellular enzymes. However, in the presence of ATP target molecules, the DNA/MoS_2_ structure disassembled to induce the release of DOX and cell apoptosis (Figure 15). As such, the authors present a drug release system, responsive to stimuli, for targeted chemotherapy, which may have other applications in the wider nanotechnology applications.

### 2.6 DNA‐NPs as Drug and Gene Delivery Platforms

Liu et al. developed a novel strategy for the fabrication of a DNA‐NP for efficient delivery of sgRNA/Cas9/Antisense for targeting a tumor‐associated gene, PLK1.^[^ ^128^ ^]^ The biocompatible DNA‐NP system demonstrated efficient inhibition of tumor growth without inducing systemic toxicity. Interestingly, due to the optical properties associated with metallic NPs, the use of DNA‐NPs to facilitate the light‐triggered release of oligonucleotides has also been explored.^[^ ^129^ ^]^ Reich and colleagues functionalized hollow gold nanospheres with two types of RNA strands: one with biotin modification for cell targeting and penetration (scaffold RNA) and the other without biotin, i.e., siRNA.^[^ ^129b^ ^]^ Flexible single‐stranded RNA was utilized to achieve dense surface‐packing, followed by hybridization with the complementary RNA strand, which maximized the assembly of the targeting peptide for cellular uptake and siRNA delivery. Riley et al. demonstrated the release of siRNA from silica core/gold shell nanospheres for the release of conjugated siRNA upon excitation with either a pulsed or continuous‐wave laser (808 nm).^[^ ^130^ ^]^ The authors observed siRNA release using either continuous‐wave and pulsed irradiation; however, the latter demonstrated a higher percentage of released duplexes. On‐demand gene silencing was achieved without the use of additional chemical modifications making these siRNA‐nanoshell conjugates desirable gene delivery candidates.

**Figure 16:**
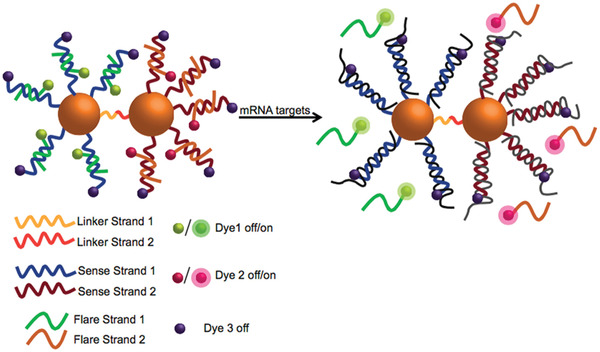
Dimer loaded with DOX (Drug 1) and mitoxantrone (Drug 2) (left). Both DOX and mitoxantrone have fluorescent properties, which are quenched due to the close proximity to the AuNP core. Drug release is achieved following binding of target mRNA to the sense strand. Target mRNA can bind to the corresponding sense sequence via competitive hybridization to induce displacement of the flare strand. Displacement is observed as an increase in fluorescence at a wavelength specific to that of the fluorophore. Reproduced with permission.^[^ ^132^ ^]^ Copyright 2018, American Chemical Society.

The use of DNA‐NPs, such as AuNPs, has also been explored as drug delivery vectors for the treatment of cancer. Heuer‐Jungemann et al. designed a NP probe that was able to accurately distinguish different cell types based on their mRNA signature.^[^ ^131^ ^]^ The probe consisted of four parts: AuNP core, a fluorophore‐tagged oligonucleotide (sense strand) attached to the AuNP, a fluorophore‐tagged oligonucleotide (flare strand) that was released from the NPs in the presence of a specific target, and finally the DNA intercalating anticancer drug (DOX). Due to the proximity of the fluorophores to the AuNP core, the fluorescence emission was quenched. However, in the presence of the specific mRNA target, the flare strand is displaced as a result of competitive hybridization, thus enabling the restoration of the fluorescence signal since it is no longer in close proximity to the gold NP core. The drug is then simultaneously released from the NPs where it can enter the cell nucleus and interfere with genomic DNA, resulting in apoptosis. Furthermore, incorporation of a fluorescent dye on the sense strands enables the DNA‐NP to serve also as a self‐reporting mechanism, ensuring that any fluorescent emission observed from the flare strand is a result of the detection of target mRNA rather than intracellular DNA degradation. More recently, the same group developed multiplexed DNA‐AuNP dimers capable of entering cells and coordinating the delivery of two different intercalating drugs, DOX, and mitoxantrone, into the local cellular environment (**Figure** **16**).^[^ ^132^ ^]^ High selectivity and specificity were achieved using oligonucleotide sequences capable of capturing a specific mRNA target. Therefore, the design of NP assemblies that can perform multiplexed synergistic roles of sensing and drug delivery activated by specific biological fingerprints within cells provides a means of improving the efficacy of therapeutic treatments by reducing nonspecific targeting and associated off‐target toxicity. The development of a DNA‐NP capable of co‐delivering nucleic acid therapeutics (short hair pin RNA) and chemotherapeutics (DOX) for the treatment of MDR breast cancer has also been reported. Following administration of the DNA‐NPs, significant inhibition of MDR1 gene expression in MDR cancer cells was achieved, which in turn resulted in enhanced intracellular accumulation of DOX and revoked drug resistance. Inhibition of tumor growth in a xenograft MDR solid tumor model was also observed.^[^ ^133^ ^]^

**Figure 17:**
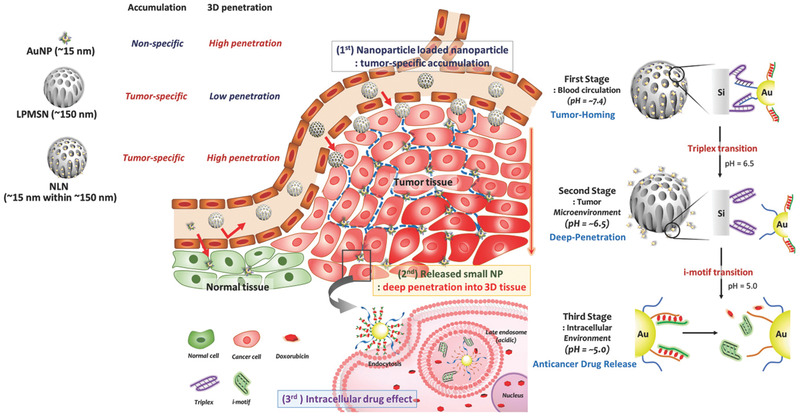
Illustration of the sequential events of the NP‐loaded NP carriers comprising the prepared large‐pored mesoporous silica NP and drug‐loaded DNA‐gold NPs (AuNPs). Loading into the pores and stimulus‐responsive release of the small AuNPs in the intracellular environment were programmed by DNA hybridizations. Reproduced with permission.^[^ ^135^ ^]^ Copyright 2018, WILEY‐VCH.

**Figure 18:**
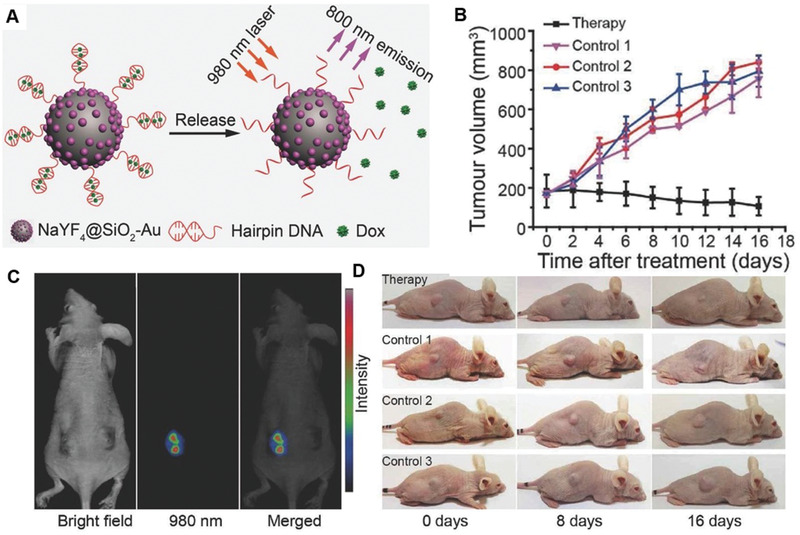
a) Schematic illustration of DOX drug release from hairpin‐DNA‐modified NaYF4@SiO_2_–Au nanoconjugates triggered by a photon 980 nm excitation. b) Changes to tumor volume following response to either of the four treatments where the three control groups indicate: only laser irradiation (control 1), without laser irradiation and treatment of nanoconjugates (control 2), and with treatment of DOX‐loaded DNA‐NPs without irradiation (Control 3). c) In vivo up‐conversion imaging of a tumor‐bearing mouse treated with the DNA_NPs. d) Pictures of the tumor bearing mice following treatment with therapy or controls. Reproduced with permission.^[^ ^138^ ^]^ Copyright 2017, WILEY‐VCH.

Stimuli‐responsive DNA‐NPs have also shown promise as drug delivery platforms. Song et al. developed a pH‐responsive DNA‐AuNP nanocarrier for the delivery of DOX to cells at a pH of less than 5.5.^[^ ^134^ ^]^ Kim et al. evaluated the use of “DNA‐functionalized NP‐loaded‐NPs” as a drug delivery platform.^[^ ^135^ ^]^ In this instance, silica NPs (150 nm) were loaded with DNA‐functionalized AuNPs (15 nm). DNA hybridization ensured efficient loading and drug release as a response to lower pH in the TME and intracellular environment. Such constructs were shown to offer prolonged circulation time as well as effective tumor irradiation in mouse models of metastatic breast cancer (**Figure** **17**). The use of NIR light has also been exploited for controlled drug release applications, namely, due to the fact that NIR light is capable of penetrating further into biological media in comparison to visible light. Using an NIR continuous wave (CW) or pulsed lasers, Halas et al. triggered the release of docetaxel (DTX) from DNA‐functionalized Au nanoshells with a SiO_2_ core.^[^ ^136^ ^]^ The results demonstrated higher cytotoxicity for CW versus pulsed‐laser‐induced DTX release from a DNA host. However, some nonspecific cell death was induced by the CW laser itself. DNA‐functionalized superstructures based on a “core‐satellite” architecture have also been designed for photothermal drug release applications.^[^ ^137^ ^]^ AuNRs were selected due to their high absorption cross‐section and photothermal conversion ability in the NIR region. Such design consisted of three main structural components: i) central core gold AuNR as structural scaffold, ii) DNA strands as molecular linkers, and iii) peripheral 5 nm spherical gold NPs (satellites) coated in polyethylene glycol (PEG). The presence of PEG helped to improve biological stability in serum‐containing media and reduced nonspecific interaction with other biomolecules. DOX loading and release kinetics were tuned through the use of linker strands with different designs. In comparison to the control without laser irradiation, a 2.1‐fold increase in DOX release was observed. In addition to gold AuNRs, hairpin DNA‐functionalized NaYF4‐SiO_2_ AuNPs have also been investigated for photothermal drug release applications (**Figure** **18**a).^[^ ^138^ ^]^ By combining single‐band anti‐Stokes NIR emission with the photothermal effect, the authors enabled the release of DOX in vivo (Figure 18c). This was achieved by utilizing the up‐conversion effect, which refers to the process when two or more low energy incident photons are absorbed and then emitted as one photon with higher energy. NaYF4@SiO_2_–Au nanoconjugates were designed to feature both excitation (980 nm) and emission (800 nm) bands inside the NIR window, thus enabling light penetration through depths of 8 mm in tissue phantoms. In comparison to the controls, tumors injected with the DOX‐loaded nanoconjugates, which were irradiated with a 980 nm laser, demonstrated a significant reduction in size, thus indicating successful photothermal drug release (Figure 18b,d). Therefore, by utilizing the depth penetration properties of NIR light, such nanoconjugates offer a promising approach for deep‐tissue imaging and guided therapy. Recently, Mou et al. developed an elegant strategy to co‐deliver nucleic acid to regulate drug‐resistant genes and simultaneously deliver the drug to cancer tissue. As a proof of concept, floxuridine was delivered in vivo to liver cancer mouse models.^[^ ^139^ ^]^ Relevant references with in vivo studies are summarized in **Table** **3**.

**Table 3:** Summary of DNA‐NS and DNA‐NP as drug and gene delivery platforms and their application in vivo

Reference number	Type of nanostructure	Applications
^[^ ^113^ ^]^	DNA tetrahedron with siRNA	Targeted delivery and improved serum stability of siRNA
^[^ ^124^ ^]^	DNA drug micellar NPs	Gene regulation, imaging, and chemotherapy in PTX‐resistant cell‐xenografted mice
^[^ ^133^ ^]^	c DNA–polylactide (PLA) micelles	Targeted chemotherapy and gene knockdown in subcutaneous MCF7/ADR
^[^ ^135^ ^]^	Au NP‐loaded SiO_2_ NPs	Delivery of chemotherapeutics throughout the tumor
^[^ ^138^ ^]^	Hairpin DNA‐functionalized NaYF4‐SiO_2_ AuNPs	Multimodal imaging and photothermal DOX release

## 3 The Clinical Translation of DNA‐NSs and DNA‐NPs

Current methods for treating cancer typically involve surgery in combination with chemotherapy and/or immunotherapy and radiation therapy. However, traditional therapies, specifically small molecule chemotherapeutics such as cisplatin, are associated with off‐target effects and lack of tumor specificity. This translates into major side effects that not all patients can tolerate, limiting the recommended therapeutic dose that can be administered in order to elicit a therapeutic effect.^[^ ^140^ ^]^ In addition, cancer is associated with a high degree of molecular heterogeneity between cancers of the same type, as well as between the primary tumor and its metastatic foci within the same patient.^[^ ^141^ ^]^ Because of these known problems, major efforts have focused on developing more personalized therapies with the hope of increasing treatment efficacy while minimizing unwanted side effects.^[^ ^8^ ^]^ Nanoformulations such as liposomes have several clinical advantages over traditional therapies including, i) reduced renal and/or hepatic circulation, ii) a greater ability to accumulate at the side of interest, and iii) less off‐target accumulation which in turn reduces systemic side effects.^[^ ^18^, ^142^ ^]^ Doxil, the first FDA‐approved nanomedicine, enabled the delivery of DOX to tumor sites via passive targeting by liposomal encapsulation.^[^ ^142^, ^143^ ^]^ As it stands, the FDA has approved numerous nanomedicines with several more currently being investigated in preclinical and clinical studies.^[^ ^142^, ^144^ ^]^ However, several drug‐delivery nanoparticle systems are a mixture of differently sized molecules, a result of the difficulties in precisely controlling the size, shape, and placement of the molecules within the nanostructure itself.^[^ ^145^ ^]^ Moreover, scaled‐up production of many nanoformulations is often challenging and is associated with high costs.^[^ ^145^, ^146^ ^]^ On the other hand, the strict base pairing principle associated with DNA enables flexible structural manipulation in the nanometer regime at a potentially lower cost.^[^ ^31^, ^145^, ^146^, ^147^ ^]^ This has resulted in the fabrication of monodisperse, highly controllable drug delivery platforms to enable control of the shape and positioning of units on the surface of DNA.^[^ ^31b^ ^]^ Furthermore, owing to its already prominent presence in nature, DNA offers high biocompatibility and low cytotoxicity and, combined with its high drug loading and theranostic capabilities, DNA‐NSs and DNA‐NSs offer many advantages over current clinically approved nanomedicines.^[^ ^31^, ^145^, ^147^ ^]^

Encouraged by the success of preclinical research, which validated the effectiveness of DNA‐NPs for the treatment of cancer,^[^ ^96a^ ^]^ significant work has focused on translating these nanostructures into the clinic.^[^ ^96^, ^98^, ^148^ ^]^ Exicure Inc., a clinical stage biotech company, utilizes spherical nucleic acid (SNA) architecture to enable safe and effective delivery of therapeutics into cells and tissues. The first clinical trial (NCT03010046) utilizing this technology investigated the use of SNA AST‐005 for the treatment of chronic psoriasis by targeting tumor necrosis factor alpha (TNF*α*), an inflammatory cytokine.^[^ ^149^ ^]^ The topically applied gel formulation was shown to meet safety requirements and importantly, reduced TNF*α* mRNA expression in psoriasis plaques.^[^ ^150^ ^]^ Unfortunately, however, results from the study indicated that there was no statistically significant decrease in echolucent band thickness, a key indicator of efficacy in psoriasis patients.^[^ ^149^ ^]^

Nonetheless, Exicure is currently investigating several other SNA based DNA‐NS at both the preclinical and clinical stage as therapeutic candidates in the fields of immuno‐oncology, neurology, and dermatology.^[^ ^151^ ^]^ NA‐gold NPs (NU‐0129) capable of targeting the overexpression of Bcl2L12 are undergoing phase 1 clinical trials to evaluate safety in patients with recurrent GBM or gliosarcoma (NCT03020017).^[^ ^152^ ^]^ Results from a recent phase 0 trial indicated that the DNA‐NP, NU‐0129, was well tolerated in patients with GBM with no unexpected adverse side effects.^[^ ^153^ ^]^ In addition, initial data also indicated that that NU‐0219 is capable of crossing the BBB. AST‐008 is a toll‐like receptor 9 (TRL9) agonist designed to activate an immune response particularly when used in combination with immune checkpoint inhibitors and is currently undergoing phase 1b/2 clinical trials (NCT03086278^[^ ^154^ ^]^ and NCT03684785,^[^ ^155^ ^]^ respectively). Initial findings in patients with advanced solid tumors indicated that administration of AST‐008, either on its own or in combination with pembrolizumab, initiated cytokine and chemokine expression and immune cell activation in patient blood to a desired level.^[^ ^151^ ^]^ A phase 2 clinical trial is currently ongoing and aims to determine the recommended phase 2 dose of AST‐008 given in combination with pembrolizumab or cemiplimab in order to provide an estimation of efficacy.^[^ ^155^ ^]^

Although results from both preclinical and early clinical trials are encouraging, as with any new medicine, there are still several challenges associated with the clinical translation of DNA‐NSs and DNA‐NPs. With regards to oncology, we believe the key factor in improving their clinical efficacy lies with improving their uptake into the TME by overcoming the associated biological barriers. Although extremely challenging, research should continue to focus on i) improving the intracellular delivery of DNA‐NS and DNA‐NPs to the tumor, ii) improving stability to prevent premature degradation of DNA‐NSs and DNA‐NPs following intravenous injection, and iii) regulate and improve biodistribution and pharmacokinetic profiles of these nanomedicines to enable maximum delivery of a payload to the tumor site and improved efficacy. Of course, the need to overcome the barriers associated with the aforementioned challenges is well understood and significant effort is underway to address them.^[^ ^145^, ^156^ ^]^ Challenges include ability to ensure DNA‐NSs and DNA‐NPs maintain their stability at physiological temperature in an environment where magnesium concentration, which plays a critical role in stabilizing the DNA duplex, is decreased.^[^ ^145b^ ^]^ In addition, efforts are being made to ensure these nanoformulations evade DNA and RNA degrading enzymes and are not overwhelmed by protein corona^[^ ^150^, ^157^ ^]^ in order to enhance their intracellular trafficking and delivery to the site of interest.^[^ ^150^, ^158^ ^]^

## 4 Conclusions and Outlook

Nanotheranostics has matured to a fascinating field, where three essential abilities crucial for improved cancer therapy can be realized: i) noninvasive detection of cancer, ii) detection of cancer progression, and iii) incorporation of therapeutic modules. With regards to preclinical settings, these all‐important parameters can be assessed, as shown by the many studies described in this review. DNA‐NPs and DNA‐NSs have opened up new avenues in this direction due to intelligent design, simple functionalization strategies, and low toxicity concerns. In this review, we discussed, in a balanced fashion, the recent advancements in the applications of such nanostructures.

DNA‐NSs and DNA‐NPs, both stemming from unique self‐assembly properties of DNA, have seen immense growth in the last two decades. The tailored and customizable size, shape, and charge of DNA‐NSs and DNA‐NPs allows efficent intracellular delivery. This is in stark contrast to DNA or siRNA‐based approaches, which do not enter cells without the help of transfection agents. Another advantage of DNA‐NSs and DNA‐NPs is their ease of attachment of imaging agents or therapeutic drugs. Moreover, densely packed DNA helices can enhance the in vivo stability against DNA‐degrading enzymes, increasing the half‐life of the nanostructures and thereby increasing the chance of reaching the sites of malignancy. To further enhance the bio‐stability, DNA nanostructures can be cloaked with membranes to enhance such feature. The above reports collectively attest that both DNA‐NPs and DNA‐NSs can potentially provide an excellent design platform for the construction of all‐in‐one imaging and novel drug delivery vehicles with optimum biocompatibility and therapeutic efficacy. The programmability of DNA‐based designs enables judicious selection of nanostructures with various shapes and sizes tuned to the desired targeting paradigm, be it to optimize shape and size for passive targeting (EPR effect) or active targeting via intelligent surface functionalization strategies.

It is important to note, however, that as a field, nanotheranostic agents are not without their limitations. This is in part due to an underestimation of the effects of tumor heterogeneity, which consequently has resulted in an over simplified pharmacokinetic model in which the tumor is thought of simply as a leaky sponge that enables NP accumulation following prolonged circulation in the blood.^[^ ^7^, ^159^ ^]^ Significant efforts have therefore focused on inhibiting NP uptake by the reticuloendothelial system and tuning renal‐clearance, however, this approach neglects the importance of the effect of the vessel leakiness, blood flow, macrophage abundance and microvessel density, and variation between tumors.^[^ ^159^, ^160^ ^]^ Therefore, it is extremely important to consider all aforementioned factors when designing the ideal nanotheranostic for use in oncology, and going forward, we encourage researchers to not simply rely on the EPR effect as an effective delivery method.^[^ ^7^, ^159^ ^]^

While the existing preclinical data involving DNA‐NPs and DNA‐NSs is undoubtedly encouraging results from current clinical trials highlight that challenges remain.^[^ ^161^ ^]^ However, should these hurdles be overcome, there is a realistic hope that therapeutic efficacy can markedly improve. Emerging technologies such as immunomodulation, i.e., the use of DNA or RNA to elicit an immune response, also has the possibility to revolutionize the field of DNA nanotheranostics.^[^ ^162^ ^]^ By taking advantage of the favorable properties associated with nucleic acid scaffolds, we envisage that methods involving DNA‐immunomodulation will generate novel, effective therapies, thus allowing researchers and clinicians to modulate cell function with high precision.^[^ ^162^, ^163^ ^]^ Moreover, by incorporating other small molecules such as imaging agents and chemotherapeutics into the DNA‐origami structure, DNA‐immunomodulation has significant potential to drive the advancement of DNA‐NSs and DNA‐NPs as theranostic tools. With further refinements of the nanostructure concepts described in this review, it is expected that cutting‐edge materials will be translated into the clinics where they are poised to improve the management and outcome of cancer patients.

## Conflict of Interest

The authors declare no conflict of interest.

## Supporting information

Supplemental Video 1

Supplemental Video 2

Supplemental Video 3

Supporting Information
