# Supplementary material for: DNA Nanostructures and DNA‐Functionalized Nanoparticles for Cancer Theranostics
Source: Adv Sci (Weinh). 2020 Oct 15;7(23):2001669. doi: 10.1002/advs.202001669 (PMC7709992; doi:10.1002/advs.202001669)
Supplement: Supplementary file 4 — Supporting Information [file ADVS-7-2001669-s001.pdf]

© 2020 Wiley-VCH GmbH

ADVANCED SCIENCE
Open Access

Supporting Information

for *Adv. Sci.,* DOI: 10.1002/advs.202001669

DNA Nanostructures and DNA-Functionalized Nanoparticles for Cancer Theranostics

*Fay Nicolson, Akbar Ali, Moritz F. Kircher, and Suchetan Pal**
